# The Learning Curve for a Fetal Cardiac Intervention Team

**DOI:** 10.1155/2010/674185

**Published:** 2010-03-30

**Authors:** Stephen P. Emery, Jacqueline Kreutzer, Frances M. McCaffrey, Fredrick S. Sherman, Hyagriv N. Simhan, Bradley B. Keller

**Affiliations:** ^1^Department of Obstetrics, Gynecology and Reproductive Sciences, Magee-Womens Hospital of the University of Pittsburgh Medical Center, Pittsburgh, PA 15213, USA; ^2^The Children's Hospital of Pittsburgh of UPMC, 15224, USA; ^3^The Department of Pediatric Cardiology, The Magee-Womens Research Institute, 15213, USA; ^4^The Division of Pediatric Heart Research Cardiovascular Innovation Institute, University of Louisville, KY 40202, USA

## Abstract

*Objectives*. Multiple technical difficulties are encountered when a multidisciplinary team of subspecialists begins a minimally-invasive fetal cardiac interventional program. We describe the learning curve. *Study Design*. Ten pregnant sheep underwent ultrasound-guided balloon valvuloplasty of the aortic valve. Team members and their roles remained constant through the trial. The time between needle insertion and entrance of the left ventricle at the aortic root was recorded. *F*-test was used to assess significance (*P* ≤ .05). *Results*. The time required to accurately position the needle tip at the aortic root decreased significantly over the course of the trial, from 12 minutes with the first attempt to one minute with the last (*P* = .003). *Conclusion*. A significant learning curve is encountered when a multidisciplinary team begins a minimally-invasive fetal cardiac intervention program. However, technical proficiency can be achieved with practice. Institutions interested in developing such a program should consider practice in an animal model before proceeding to the human fetus.

## 1. Introduction

Balloon valvuloplasty for fetal critical aortic stenosis has been shown to alter the developmental trajectory of some fetuses from hypoplastic left heart syndrome (HLHS) toward restored left ventricular growth [1–4]. Technical success holds the promise of two ventricle circulation in the neonate, thereby avoiding the need for three-stage palliation to single ventricle circulation or cardiac transplant [4]. The fact that this can be achieved through a single needle insertion through the maternal abdomen into the fetal chest is especially appealing in that it avoids the profound morbidity associated with the diagnosis of HLHS in the neonate, while exposing mother and fetus to minimal risk. There are multiple technical challenges involved in performing fetal valvuloplasty. Some of these difficulties have to do with communication among team members during the navigation of the needle through the maternal abdomen, uterine wall, fetal chest wall, and into the left ventricle pointing directly at the aortic root. While learning to function as a team, the members of the interventional program must agree on a standard orientation for ultrasound imaging that takes into consideration the needle trajectory and target. Finally, complications occur during fetal cardiac intervention such as arrhythmia, hemopericardium, and cardiac tamponade that require a rapid and coordinated response. Use of a large animal training paradigm, typical for surgical training on medical device implantation, could allow the team to learn the technique, develop effective communication, and practice appropriate responses to complications. 

We describe the learning curve encountered by a multidisciplinary team of maternal-fetal medicine, interventional cardiology, and fetal echocardiography subspecialists during the initiation of a minimally-invasive fetal cardiac intervention program. Our aim is to demonstrate that technical challenges are significant but are surmountable. Practice in a large animal training model should be considered before proceeding to the human fetus.

## 2. Materials and Methods

### 2.1. Study Design

 This study is a secondary analysis of a trial comparing a computer-assisted navigational system (CANav) to ultrasound-guided “freehand” insertion of an operative needle into the left ventricle of the sheep fetus. In that study, the time required to navigate the needle from the maternal abdomen into the left ventricle using CANav was compared to the time required to perform a similar function without. The present study describes the time interval during the “freehand” attempts over the course of the trial, and represents the learning curve of the multidisciplinary team.

### 2.2. Equipment

 Based on the published methods for human fetal cardiac intervention [4–8] and personal experience, we selected needles, wires, and catheters currently in use in the human fetus. 

A standard thin-walled Cook 19-gauge intravascular needle (DTN-19UT-11.5-M3, Cook International, Bloomington, IN) was used. This needle contains a solid trocar with an axi-symmetric, sharp tip and permits smooth passage of a 0.014 inch diameter guide wire. An 18-gauge Cook interventional needle with a unique external Echotip markings to enhance ultrasound visualization (G05045, 15 cm length, Hawkins-Atkins blunt tip needle, Cook International, Bloomington, IN) was also evaluated. We used either a standard floppy-tip 0.014′′ guide wire (22339M, Floppy II, Guidant Corporation) or a tip-deflecting guide wire (STEER-IT 535DSM014, Cordis Corporation, Miami Lakes, FL). Finally, a standard 3 mm outer diameter coronary angioplasty balloon (H7492062009300, 3.0 mm balloon diameter, 9 mm balloon length, Maverick Over-The-Wire, Boston Scientific, Natick, MA or 563-FX030, 3.0 balloon diameter and 10 mm balloon length, Ninja FX, Cordis Corporation, Miami Lakes, FL) was used for the procedures.

### 2.3. Animal Trials

 All experiments using pregnant sheep were performed under an approved Institutional Animal Care and Use Protocol (no. 0612581) of the University of Pittsburgh. We studied 10 pregnant ewes, two with twins (*n* = 12 fetal sheep) at between 85 and 110 days of a 145-day gestation [9].The newborn lamb and human have comparable average weights (3.5 kg) and we selected a period in fetal lamb gestation when the average fetal lamb aortic valve annulus and average left ventricular long axis length are comparable to the 20-to-30 week gestation human fetus. Pregnant ewes arrived from the supplier approximately 3 days before the procedure and a complete physical examination was performed prior to anesthesia and instrumentation.

The research team for the fetal sheep cardiac intervention trials included (1) a maternal-fetal medicine specialist with intrauterine intervention experience, (2) a veterinary anesthetist with 2 support staff, (3) 2 pediatric cardiologists trained in fetal cardiology, (4) a pediatric cardiologist who specializes in technology development, and (5) a pediatric cardiologist with interventional cardiology expertise. Prior to the start of each procedure, the team reviewed the interventional technique and the role each member was to play.

Maternal body temperature was monitored using a digital rectal probe and maintained between 36°C and 37°C, using a warming blanket. Heart rate was monitored using standard ECG electrodes and module. Pregnant sheep were sedated with Ketamine (10 mg/kg i.v.), intubated for mechanical ventilation, and then anesthesia was continued with isoflurane (2%) and 100% oxygen. Pavulon (0.1 mg/kg i.v.) was given to suppress spontaneous movement. Fluid-filled intravascular catheters were placed in the carotid artery via cut down to measure arterial pressure and withdraw blood samples and in the jugular vein to infuse fluids. Intermittent blood samples were withdrawn to measure blood gas content for acid-base status as an index of adequacy of ventilatory support.

The maternal lower abdominal fur was shaved and prewarmed ultrasound coupling gel was used for ultrasound imaging. Each sheep fetus was imaged using a clinical ultrasound system (HD11, Phillips Medical Systems, Bothwell, WA) and a curved array obstetrics ultrasound probe (C5-2, Phillips Medical Systems, Bothwell, WA). A general screening fetal ultrasound was performed to identify the presence of a single or twin gestation and to determine the orientation of each sheep fetus within the maternal abdomen. Optimizing fetal positioning to allow for a percutaneous approach was attempted transabdominally. If unsuccessful, laparotomy was performed followed by uterine manipulation in order to properly position the fetus (left side up). Time required to position the fetus was not included in the trial. Prior to starting the procedure, a single dose of atropine (1 mg/kg) was delivered to the fetal thigh as an intramuscular injection to prevent fetal bradycardia. 

Fetal cardiac ultrasound was performed to determine the fetal heart rate, left ventricular short-axis and long-axis dimensions (mm), aortic valve annulus diameter (mm), and the presence or absence of pericardial fluid. The midpoint of the aortic valve and the left ventricular (LV) apex were determined using alternating long-axis and four-chamber views. These two points define an optimal trajectory for the interventional needle path. Following a 2 mm surgical skin incision, the needle was advanced through the maternal skin and uterine wall into the amniotic cavity under ultrasound visualization. The trajectory of the trocar and needle were continuously updated and displayed on the ultrasound image. After confirming that the needle trajectory remained “on-path”, the needle was then advanced through the fetal chest wall, fetal pericardium, fetal ventricular apex and into the left ventricular cavity (Figure 2). Once the needle was positioned within the LV cavity, the trocar was removed and 0.014′′ guide wire and premounted coronary balloon were advanced across the aortic valve (Seldinger technique) to perform balloon valvuloplasty. The balloon catheter was then inflated during ultrasound imaging (Figure 3). Following balloon deflation, the balloon and wire were withdrawn through the needle taking care to maintain the tip of the needle within the fetal left ventricle. Fetal intracardiac atropine (0.1 mg/kg) was given via the needle if bradycardia developed during the procedure.

### 2.4. Laparotomy

 If an acceptable trajectory was not available due to an unfavorable fetal position, a small lateral laparotomy incision was performed to allow manual repositioning of the fetus within the uterus and/or expose the uterus for direct trocar and needle puncture. This surgical procedure involved a vertical incision through the skin and subcutaneous tissues to reach the peritoneal cavity. Vascular structures were cauterized or ligated if transected. The peritoneum was then opened to visualize the uterus. Warmed, saline-soaked sponges were used to pack the intestine from the operative field. Sterile ultrasound gel and a sterile ultrasound transducer sheath were used on the uterine surface to allow ultrasound imaging of the fetus. 

All attempts to enter the fetal left ventricle (LV) were successful. The time between initiation of needle insertion and entrance of the LV at the aortic root was recorded as a composite measure of team communication and decision-making, and was the time interval recorded in the computer-assisted navigation trial. Time to balloon inflation was not recorded independently of needle insertion as it was felt to be independent of the navigational component of the procedure. In order to maximize use of the animals, several attempts were made per animal. The time interval between induction of anesthesia and needle insertion (with or without laparotomy) was not recorded. Only attempts without computer-assisted navigation were analyzed. *F*-test was used to assess a significant difference in the time to complete this complex set of tasks (initial trial versus final trial, *P* ≤ .05).

## 3. Results

**Figure 1:**
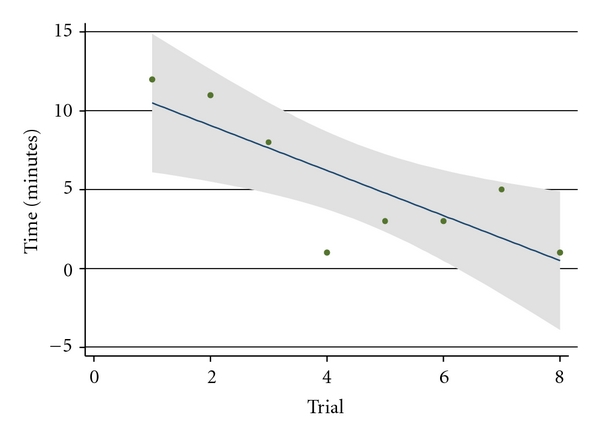
Needle navigation time versus trial number. The time required to accurately position the needle tip at the aortic root decreased significantly over the course of the trial, from 12 minutes with the first attempt to one minute with the last. *P* = .003, *F*-test.

**Figure 2:**
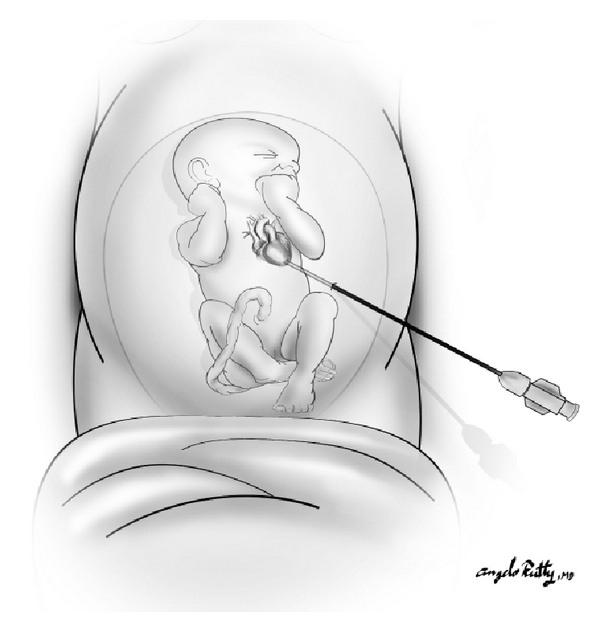
Illustration of the correct needle trajectory through the maternal abdomen and into the fetal left ventricle at the apex.

**Figure 3:**
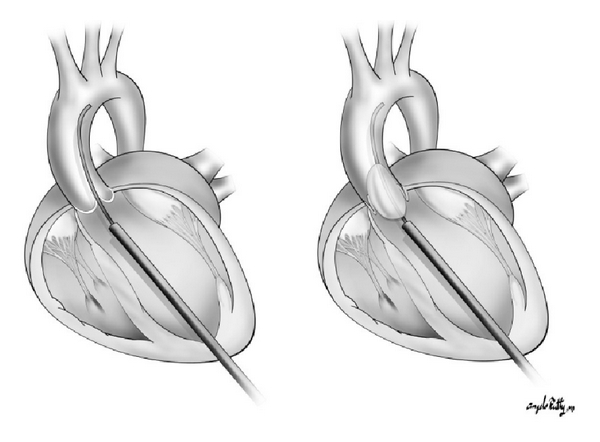
Illustration of the correct needle placement to allow for wire passage and balloon inflation across the aortic valve.

The time required to traverse the uterus, enter the fetal chest, and accurately position the needle tip at the aortic root decreased significantly over the course of the trial, from 12 minutes with the first attempt to one minute with the last (*P* = .003) (Table 1 and Figure 1). The time decreased by 0.54 minutes per trial.

Five of ten sheep (50%) required laparotomy for fetal repositioning. Animals 2, 3, 6, 7, and 10 underwent both computer-assisted and unassisted trials in an effort to maximize use of the animal.

## 4. Comments

Our study was designed to simulate, for training and equipment validation purposes, human fetal cardiac intervention as it is currently performed. The animals were surgically prepped, sterile technique was used in a modern operating suite, equipment is identical, and personnel involved in the trials were those who will perform human fetal interventions. As the location, equipment and personnel remained relatively constant throughout the trial; our results demonstrate the learning curve of the team and not that of any one individual.

There is a significant learning curve encountered when a multidisciplinary team of subspecialists begins a fetal cardiac intervention program. The learning curve results from a number of obstacles. First, members the group are not necessarily used to working together during an invasive procedure. Communication, teamwork, and cooperation take time to develop. 

Second, a standard ultrasound orientation is necessary for accurate sonographic guidance during the procedure. For instance, some fetal echocardiographers invert and/or flip the ultrasound image to mimic the orientation of pediatric echocardiography. Obviously, this can lead to significant confusion among team members not familiar with this orientation. 

Third, unlike other ultrasound-guided intrauterine procedures, the needle trajectory in fetal cardiac intervention must be precise. The target is fixed within the maternal abdomen and is approachable by only one narrow trajectory in *X*, *Y*, and *Z* planes. We therefore developed a method whereby the ultrasound probe is viewed in relation to the face of a clock, where the reference indicator is 12:00; the 3:00, 6:00, and 9:00 positions logically follow. This allows for ultrasound guidance only in relation to the ultrasound probe and target and allows for continuous updating of the needle path with the desired trajectory. Arbitrary and confusing terms such as “more toward the left” are avoided. This technique allows for accurate sonographic guidance irrespective of the relation between the desired trajectory, the maternal abdomen, and the fetal lie.

A fourth obstacle is fetal position. As with all surgery, adequate exposure and positioning are essential to successful intervention. Successful cardiac intervention requires precise placement of the needle tip pointing directly at the aortic root. If an adequate trajectory cannot be established, the procedure will fail. Although it is tempting to attempt needle placement percutaneously, maternal laparotomy with fetal repositioning will more likely ensure a successful procedure. Additionally, a failed percutaneous attempt can result in fetal compromise, rendering subsequent attempts more difficult. Experience allows the development of judgment on whether a percutaneous approach is achievable, which was a major component of our learning curve. Overall, 50% of our sheep required laparotomy for fetal repositioning, similar to the rate reported elsewhere [4, 10]. 

Management of these obstacles contributes to efficient and successful catheter placement. We therefore chose the interval between initiation of the trajectory and needle placement in the left ventricle as a measure of teamwork, communication and judgment. We elected to not analyze catheter attempts using computer-assisted navigation, as institutions developing interventional programs are unlikely to have access to such technology. Even with computer-assisted navigation, the main obstacles remain. 

Finally, as with all surgical interventions, complications will occur. Some of the complications our team encountered included arrhythmias, hemopericardium, cardiac tamponade, and equipment failure. Having witnessed and managed these events in the animal model is likely to improve their successful management in the human fetus. 

There are several limitations to our study. The first is that it is a secondary analysis of a validation study which compared the success of computer-assisted navigation to that of “freehand” fetal cardiac intervention [8]. Analysis of the learning curve was not the primary intent of the study. Therefore, certain time intervals and outcomes that may have supplemented this manuscript were not recorded. A second limitation is fewer data points for time to LV without navigation (*n* = 8) compared to that of with navigation (*n* = 16). A third is that we used the same team for all of the experiments. A comparable approach using the same paradigm and a different team would provide additional validation. Finally, we could not randomize the gestational age of the fetuses during the study. It is possible that fetal gestation was an important determinant of the time to complete intervention. 

 In summary, a significant learning curve is encountered when a multidisciplinary team of obstetric and pediatric subspecialists begins a minimally-invasive fetal cardiac intervention program. We advocate that institutions interested in developing a cardiac intervention program should consider practice in an animal model before proceeding to the human fetus.

## Figures and Tables

**Table 1:** Table summarizing trial number, week of the trial, animal number, fetal position, need for laparotomy, and the time required to navigate the needle to the correct position in the left ventricle. The original computer-assisted navigation investigation involved 24 trials. Of these, 8 were done “freehand, ” that is, without computer-assisted navigation. These 8 trials are used in the current analysis.

Trial no.	Week no.	Animal no.	Position	Lap YES/NO	Time to LV w/o Navigation
5	2	2 twin A	Transverse	Yes	12
6	2	2 twin B	Transverse	Yes	11
10	3	3	Cephalic	No	8
16	4	6	Cephalic	No	1
18	4	7	Cephalic	No	3
20	4	8	Cephalic	No	3
21	4	8	Cephalic	No	5
24	5	10	Transverse	Yes	1

## References

[1] Tobita K, Keller BB (2000). Right and left ventricular wall deformation patterns in normal and left heart hypoplasia chick embryos. *American Journal of Physiology*.

[2] Hornberger LK, Sanders SP, Rein AJ, Spevak PJ, Parness IA, Colan SD (1995). Left heart obstructive lesions and left ventricular growth in the midtrimester fetus: a longitudinal study. *Circulation*.

[3] McCaffrey FM, Sherman FS (1997). Prenatal diagnosis of severe aortic stenosis. *Pediatric Cardiology*.

[4] Tworetzky W, Wilkins-Haug L, Jennings RW (2004). Balloon dilation of severe aortic stenosis in the fetus: potential for prevention of hypoplastic left heart syndrome. candidate selection, technique, and results of successful intervention. *Circulation*.

[5] Marshall AC, van der Velde ME, Tworetzky W (2004). Creation of an atrial septal defect in utero for fetuses with hypoplastic left heart syndrome and intact or highly restrictive atrial septum. *Circulation*.

[6] Marshall AC, Tworetzky W, Bergersen L (2005). Aortic valvuloplasty in the fetus: technical characteristics of successful balloon dilation. *Journal of Pediatrics*.

[7] Wilkins-Haug LE, Tworetzky W, Benson CB, Marshall AC, Jennings RW, Lock JE (2006). Factors affecting technical success of fetal aortic valve dilation. *Ultrasound in Obstetrics and Gynecology*.

[8] Emery SP, Kreutzer J, Sherman FR (2007). Computer-assisted navigation applied to fetal cardiac intervention. *International Journal of Medical Robotics and Computer Assisted Surgery*.

[9] Rudolph AM (2000). Myocardial growth before and after birth: clinical implications. *Acta Paediatrica*.

[10] Mäkikallio K, McElhinney DB, Levine JC (2006). Fetal aortic valve stenosis and the evolution of hypoplastic left heart syndrome: patient selection for fetal intervention. *Circulation*.

